# Engineering probiotics as living diagnostics and therapeutics for improving human health

**DOI:** 10.1186/s12934-020-01318-z

**Published:** 2020-03-04

**Authors:** Zhao Zhou, Xin Chen, Huakang Sheng, Xiaolin Shen, Xinxiao Sun, Yajun Yan, Jia Wang, Qipeng Yuan

**Affiliations:** 1 State Key Laboratory of Chemical Resource Engineering, Beijing University of Chemical Technology, 15# Beisanhuan East Road, Chaoyang District, Beijing, 100029 China; 2 College of Engineering, The University of Georgia, Athens, GA 30602 USA

**Keywords:** Probiotics, Metabolic engineering, Synthetic biology, Microbiome

## Abstract

The gut microbiota that inhabit our gastrointestinal tract are well known to play an important role in maintaining human health in many aspects, including facilitating the digestion and absorption of nutrients, protecting against pathogens and regulating immune system. Gut microbiota dysbiosis is associated with a lot of diseases, such as inflammatory bowel disease, allergy, obesity, cardiovascular and neurodegenerative diseases and cancers. With the increasing knowledge of the microbiome, utilization of probiotic bacteria in modulating gut microbiota to prevent and treat a large number of disorders and diseases has gained much interest. In recent years, aided by the continuous development of tools and techniques, engineering probiotic microbes with desired characteristics and functionalities to benefit human health has made significant progress. In this paper, we summarize the recent advances in design and construction of probiotics as living diagnostics and therapeutics for probing and treating a series of diseases including metabolic disorders, inflammation and pathogenic bacteria infections. We also discuss the current challenges and future perspectives in expanding the application of probiotics for disease treatment and detection. We intend to provide insights and ideas for engineering of probiotics to better serve disease therapy and human health.

## Background

The human gastrointestinal tract harbors complex and diverse microbes that act as a key factor in maintaining the homeostasis of the intestinal microenvironment [1]. It is estimated that approximately 10^13^–10^14^ bacterial cells from more than 1000 different species are present in gut, which form a natural ecosystem in the human body [2]. Those commensal bacteria can utilize the nutrients in the gut to produce metabolites to form a host-microbe metabolic axes [3]. Within those metabolic axes, it is able to regulate nutrient absorption, energy metabolism and various physiological processes in host [4]. Recently, study of interaction between gut flora and human host has gained much interest. More and more evidence indicated that gut microbiota plays an important role in human health and diseases [5]. On the one hand, the human gut microbiota contributes to supply essential healthy nutrients, digestion of food, reduction of inflammation and breakdown of toxins, promotes hematopoiesis and enteric nerve function, and regulates the host’s immune system [6]. On the other hand, the abnormal changes of the gut ecosystem are associated with pathological conditions such as atherosclerosis, hypertension, heart failure, chronic kidney disease, obesity, diabetes and cancer [7–15]. In addition, the composition of the gut microbiota in humans can indicate the disease risk or development [16, 17]. It has been reported that diet can significantly alter the gut microbiota composition, other factors including host genetics, infections, the use of antibiotics, immunosuppressive therapy and other means of treatment also contribute to the composition and effect of the gut flora [18, 19].

Probiotics are live bacterial species that are able to survive and thrive in the acidic gastric environment and provide beneficial effect on the health of the host by reestablishing or improving the gut microbiota [20]. Introduction of probiotics into the gastrointestinal tract might serve as a promising strategy to restore the balance of gut ecosystem and prevent or treat illness. It has made great progress in employment of a living product rather than a chemical to diagnose or treat diseases including diabetes, phenylketonuria, HIV and inflammatory bowel disease (IBD) [21–24]. However, there is no one-size-fits-all probiotic that works well for everyone as the gut microbiome is different in individuals. With the development of metabolic engineering and synthetic biology, engineering of probiotics opens up possibilities of designing microbes to target specific tissues and cells rather than the whole body and creation of novel probiotics with desired characteristics and functionalities. In this review, we summarize the recent advances in engineering probiotic bacteria as living diagnostics and therapeutics for probing and treating metabolic disorders, inflammation and pathogenic bacteria infections (Table 1).

**Table 1:** A summary of the applications for engineered probiotics

Condition	Probiotic used	Animal model	Administration	Refs.
Diabetes	*L. lactis*	Mice	Oral	[35, 36]
	*L. lactis*	Rats	Oral	[39]
	*L. gasseri*	Rats	Oral	[22]
Phenylketonuria	*E. coli* Nissle 1917	Mice/monkeys	Oral	[28]
Hyperammonemia	*L. helveticus*	Rats	Oral	[47]
	Gut microbiota of murine	Mice	ASF	[48]
	*E. coli* Nissle 1917	Mice	Oral	[29]
*P. aeruginosa* infection	*E. coli*	In vitro	None	[63, 64]
	*E. coli* Nissle 1917	Mice	Oral	[57]
EHEC/*S. aureus*/*S. epidermidis* infection	*E. coli* Nissle 1917	In vitro	None	[62]
*Salmonella* infection	*E. coli* Nissle 1917	Mice	Oral	[68]
	*E. coli* Nissle 1917	In vitro	None	[61]
	*E. coli* Nissle 1917	Turkey	Oral	[71]
*V. cholerae* infection	*E. coli* Nissle 1917	Mice	Oral	[75, 76]
	*L. lactis*	Mice	Oral	[77]
	*V. cholerae*	Rabbit	Oral	[78]
	*L. lactis*	Mice	Oral	[77]
Liver metastasis	*E. coli* Nissle 1917	Mice	Oral	[87]
Gut inflammation	*E. coli*	In vitro	None	[92, 97]
	*E. coli* Nissle 1917	Mice	Oral	[98]

### Engineering probiotics for amelioration of metabolic disorders

**Fig. 1:**
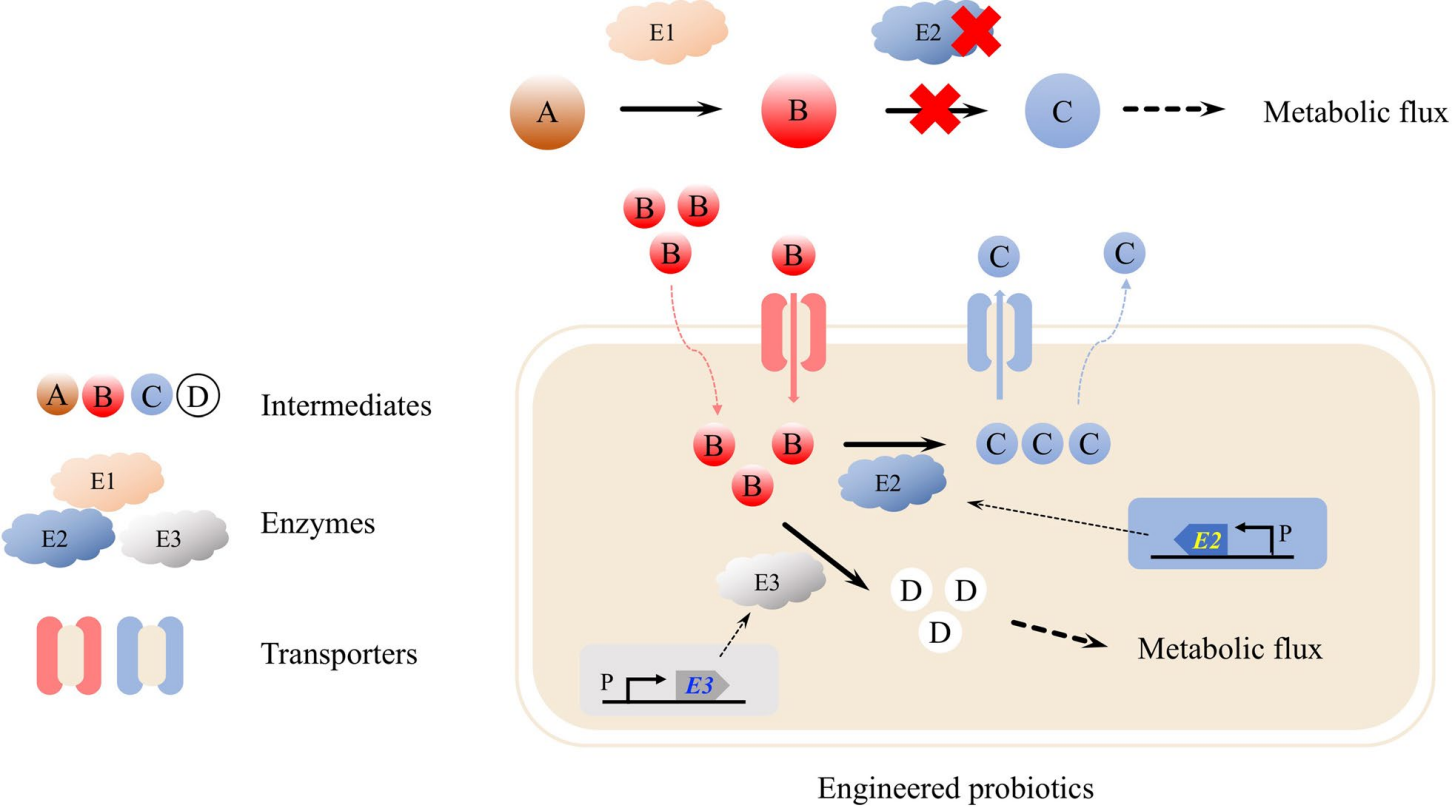
Engineering probiotics for the treatment of metabolic disorders caused by enzyme deficiency. The lack of E2 results in the accumulation of intermediate B and the insufficient supply of intermediate C. The engineered probiotics harboring E2 and/or E3 can be used to restore metabolism and eliminate the accumulation of the intermediate B

Enzymes play a central role in cellular metabolism and catalyze complicated biological processes to maintain life. The steady state of the metabolism depends upon multiple enzymatic reactions which can be interrupted by enzyme deficiency [25]. The missing or defective enzyme results in metabolic disorders in which toxic metabolites may accumulate or essential products may not be produced [26, 27]. Taking enzyme replacements to restore metabolism and removing toxic products or inhibiting their synthesis are promising treatments for metabolic disorders (Fig. 1). Many researches have suggested that engineered probiotics harboring specific enzymes or pathways are able to relieve metabolic disorders in organisms [28, 29].

Diabetes is a disease in which blood glucose levels in the body rise higher than normal due to the pancreas is unable to produce sufficient hormone insulin. This can result in a variety of complications such as cardiovascular and Alzheimer’s diseases, stroke and nerve damage [30]. Type I diabetes (T1D) is an autoimmune disease in which the immune system attacks and impairs cells in the pancreas. While, type II diabetes (T2D) is generally caused by the insulin resistance [31]. Currently, the use of insulin and hypoglycemic drugs and cytokine-based therapeutics are the main and effective treatments for diabetes [32–34]. Compared with these traditional therapeutics, utilization of probiotics for diabetes treatment has less side effects and is able to avoid the pain caused by injection. Recently, the researchers engineered a human gut strain *Lactococcus lactis* for the treatment of T1D. The modified *L. lactis* was capable of secreting whole proinsulin autoantigen and biologically active immunoregulatory cytokine interleukin-10 (IL-10). The combination therapy with oral administration of recombinant *L. lactis* and low-dose of nonspecific immune modulator anti-CD3 was employed to test whether the non-obese diabetic (NOD) mouse could revert normoglycemia after diabetes onset. The results shown that 59% of animal models (36 out of 61 mice) had a stable recovery of autoimmune diabetes compared with the control group [35]. In another example, Robert et al. engineered *L. lactis* to secrete the T1D autoantigen GAD65370–575 and the cytokine IL-10. In combination with short-course low-dose anti-CD3, this treatment can stabilize the pancreas islet inflammation in NOD mice even in the case of severe hyperglycemia [36]. Glucagon-like peptide (GLP) 1 is a regulator for various homeostasis events. It is produced by post-translational processing of proglucagon [37]. It has been reported that GLP-1-(1–37) is able to induce insulin production both in vivo and in vitro by converting adult intestinal epithelial cells into functional insulin-producing cells [38]. On this basis, Duan et al. designed a recombinant strain *L. gasseri* for secretion of GLP-1-(1–37) to reduce blood glucose levels. The gene encoding GLP-1-(1–37) was fused with a USP45-LEISS secretion marker (SEC) and a polyhistidine (HIS) tag, separated by an enterokinase site. This expression cassette under control of the promoter *P*_*slpA*_ was inserted into the chromosome of *L. gasseri*. The in vitro results indicated that GLP-1-(1–37) could convert rat cells into insulin-secreting cells. When feeding recombinant *L. gasseri* into diabetic rats, a large number of insulin-producing cells was developed in the upper intestine, the numbers is sufficient to replace 25–33% of the insulin capacity of non-diabetic healthy rats [22]. In addition, *L. lactis* harboring GLP-1 was also employed for the treatment of T2D. It has been observed that once engineered *L. lactis* carrying a plasmid vector encoding GLP-1 was orally administered in Zucker diabetic fatty (ZDF) rats, secretion of insulin was significantly stimulated from a pancreatic beta cell line HIT-T15, and the blood glucose levels were decreased by 10–20% during 2–11 h post dosing [39]. Although engineering of bacteria for the treatment of diabetes have made great progress in animal models, there have been very few clinical trials reported so far.

Phenylketonuria (PKU) is a genetic metabolic disorder that prevents patients from breaking down the amino acid phenylalanine [26]. It is caused by a defect in the phenylalanine hydroxylase (PAH) which is an enzyme responsible for converting the phenylalanine into other metabolites. Accumulation of phenylalanine at abnormally high levels in the blood could result in serious health issues including emotional and behavioral difficulties, seizures, tremors and marked intellectual disability [40]. Phenylalanine ammonia lyase (PAL) is able to convert phenylalanine into ammonia and *trans*-cinnamic acid, which serves as a potential enzyme for PKU therapy [41]. A large amount of PAL protein was obtained by heterologous over-expression of *PAL* gene from *Rhodosporidium toruloides* in *Escherichia coli.* Administration of PAL to the *PAH*^enu2^ mouse model of human PKU successfully lowered the plasma phenylalanine concentration [42]. In another study, the metabolically engineered *Lactobacillus reuteri* carrying *PAL* from *Anabaena variabilis* could greatly reduce blood phenylalanine in the mice model of PKU within 4–5 days of treatment. It also observed that the probiotic microbe was lost from the intestine at 8 months post treatment [43]. Recently, an engineered probiotic SYNB1618 was constructed for degradation of phenylalanine. In this case, PAL and L-amino acid deaminase (LAAD), an enzyme responsible for converting phenylalanine into phenylpyruvate, were introduced into *E. coli* Nissle 1917. The programed probiotic strain can secrete those two enzymes that are activated in the gut under anoxic conditions. Oral treatment of the *PAH*^enu2/enu2^ mouse model of PKU with SYNB1618 significantly reduced the blood phenylalanine by 38% compared with the control. Additionally, administration of SYNB1618 to healthy cynomolgus monkey also inhibited the characteristic increases of plasma phenylalanine after a diet challenge [28]. This treatment is now in clinical trials (NCT03516487) to determine the effective dose in the human body.

Hyperammonemia is a metabolic disorder characterized by an excessive amount of ammonia in the blood [44]. It results from the inability of metabolizing free ammonia to urea due to the defect in enzymes and transporters participated in urea cycle [45]. Lactulose or antibiotics administrations have been suggested to treat hyperammonemia by blocking the ammonia formation in intestine or inhibiting its absorption into the body, which are not effective and suffered from many side effects [46]. Recent study has found that oral administration of probiotic *L. helveticus* strain NS8 can release the cognitive decline and anxiety-like behaviors in hyperammonemia rats, indicating that employment of the gut flora to remove the ammonia from the gut could be served as a promising treatment for hyperammonemia [47]. Shen et al. employed altered Schaedler flora (ASF), a defined consortium of eight gut commensal organisms with minimal urease effect, to treat the hyperammonemia-induced encephalopathy and neurotoxicity. Colonization of those ASF enabled the establishment of a new gut microbiome that resulted in the decrease in urease activity and ammonia generation. Transplantation of ASF to thioacetamide-treated acute and chronic hepatic injury mice successfully lowered the blood ammonia level and improved its survival rate and behavioral performance [48]. Most recently, Kurtz et al. engineered *E. coli* Nissle 1917 to generate probiotic strain SYNB1020 that is able to dramatically convert ammonia into l-arginine. In this strain, the genes *thyA* and *argR* encoding the negative regulators were deleted to activate the transcription of several genes involved in biosynthesis and transportation of arginine. Besides, the gene *argA215*, encoding a feed-back resistant *N*-acetylglutamate synthase (*argA*^fbr^) was integrated into the genome to enhance the biosynthesis of arginine. Administration of SYNB1020 reduced blood ammonia level and improved survival rate to 50% in the *spf*^ash^ mouse model of hyperammonemia. This strain was further advanced to a phase 1 clinical study and has demonstrated dose-dependent activity in hyperammonemia disorders [29]. Now, SYNB1020 has been recently moved into a phase 1b/2a clinical trials to test its safety, pharmacodynamics and tolerability (NCT03447730).

Levodopa is a primary medicine for the treatment of Parkinson’s disease [49]. After crossing the blood-brain-barrier (BBB), it can be decarboxylated into dopamine catalyzed by the aromatic acid decarboxylase (AADC) to active therapeutic activities [50]. However, levodopa also can be decarboxylated in the gastrointestinal tract to produce dopamine in the periphery, which leads to undesired intestinal side effects and reduced bioavailability [51]. Although co-administration of levodopa with carbidopa, an inhibitor of the AADC, would allow more levodopa to enter the brain by inhibiting the peripheral breakdown of levodopa, the bioavailability is no higher than 50% [52]. A recent study reported an interspecies pathway for levodopa metabolism in the human gut flora. The pyridoxal phosphate-dependent tyrosine decarboxylase (TyrDC) from *Enterococcus faecalis* and the molybdenum cofactor-dependent dopamine dehydroxylase (Dadh) from *Eggerthella lenta* were identified as two enzymes responsible for converting levodopa to *m*-tyramine in the gut. The researchers also identified a compound *S*-α-fluoromethyltyrosine (AFMT) that was able to inhibit the decarboxylation of levodopa in the gut microbial. It also observed that coadministration of AFMT with levodopa and carbidopa successfully improved the levodopa bioavailability in mice [52]. Alzheimer’s disease is a chronic neurodegenerative disorder in which the progressive loss of brain cells leads to memory loss and cognitive decline [53]. Currently, nearly all *β*-amyloid (Aβ) and tau-centric therapeutic strategies tested for Alzheimer’s disease have failed in clinical trials, which indicates the great need for development of novel therapies to treat this complicated disease. Most recently, Wang et al. observed that the progression of Alzheimer’s disease is associated with the alteration of gut flora composition. The gut dysbiosis causes the peripheral accumulation of phenylalanine and isoleucine which promotes the activation of M1 microglia, contributing to cognitive impairment. Based on this mechanism, a sodium oligosaccharide drug named GV-971 was used to harness neuroinflammation and cognitive impairment in Alzheimer’s disease progression by reestablishing gut microbiota [54]. This drug has been advanced into a phase 3 clinical study in China and the results shown that it is effective to treat Alzheimer’s disease (NCT02293915).

### Engineering probiotics for the treatment of bacterial infections

Bacterial infections are the major cause of morbidity and mortality worldwide [55]. The use of antibiotics is the mainstay of therapy to prevent and treat bacterial infections. However, the excessive use of antibiotics has contributed to antibiotic resistance, which is one of the greatest public health threats [56]. Engineering probiotics to inhibit pathogenic bacteria for the maintenance of gastrointestinal health and treatment of bacterial infections have gained much interest in recent years [57, 58]. As an innovative and alternative measure to treat infectious diseases, probiotic therapy contributes to reduction of the development of multi-resistant bacteria. It has been proposed that probiotics can inhibit pathogens by different mechanisms, including secretion of antibacterial chemicals, stimulation and modulation of the immune responses, competition of nutrition and specific adhesion sites, and inhibition of toxic protein expression in gastrointestinal pathogens [59–62]. According to the mechanistic basis, various probiotics have been developed, and most of them demonstrated great specificity and efficacy for the inhibition of pathogens.

Many chronic infections are caused by bacterial biofilm formation which leads to resistance against antibiotics and human immune system. Engineering probiotics for inhibition of biofilm formation serves as one of the mechanisms to depress pathogens [63, 64]. In a previous study, an engineered *E. coli* strain was constructed to detect and kill *Pseudomonas aeruginosa* via a quorum-sensing signaling system. In this system, the promoter P_LasI_ induced by *P. aeruginosa* quorum sensing molecule N-acyl- homoserine lactone (AHL) was introduced to drive the expression of a genetic circuit consists of the genes encoding E7 lysis protein and pyocin S5, a narrow-spectrum bacteriocin targeting *P. aeruginosa* infection. The produced E7 lysis protein is responsible for lysing the *E. coli* cells so that the generated pyocin S5 can be released into the extracellular medium to kill *P. aeruginosa* specifically. The results showed that the engineered *E. coli* can inhibit the biofilm formation by 90% and reduce the growth of planktonic *P. aeruginosa* by 99% [63, 64]. This system was further modified to prevent *P. aeruginosa* gut infection in animal models by employment of the probiotic strain *E. coli* Nissle 1917 as the host. Compared with the previous system, an extra anti-biofilm protein dispersin B was introduced to destabilize mature biofilms to achieve a more physiologically relevant treatment. The in vivo efficacy of this system was evaluated in both *Caenorhabditis elegans* and mouse infection models. It observed that the survival time of the treatment group increased more than twofold in *P. aeruginosa*-infected *C. elegans* model. In the *P. aeruginosa*-infected mouse model, the *P. aeruginosa* levels were reduced by 77% in the treatment group compared with the control group, and the probiotic can colonize in the mouse gut for up to 3 weeks [57]. Besides, probiotics also can be engineered to have an inhibitory effect on enterohemorrhagic *E. coli* strain (EHEC), *Staphylococcus aureus* and *S. epidermidis* infections. Fang et al. identified a protease DegP within the periplasm of *E. coli* Nissle 1917, it can inhibit the biofilm formation of EHEC strain. Additionally, it also demonstrated significant repression effect on the growth rates of Gram-positive pathogens *S. aureus* and *S. epidermidis* [62].

**Fig. 2:**
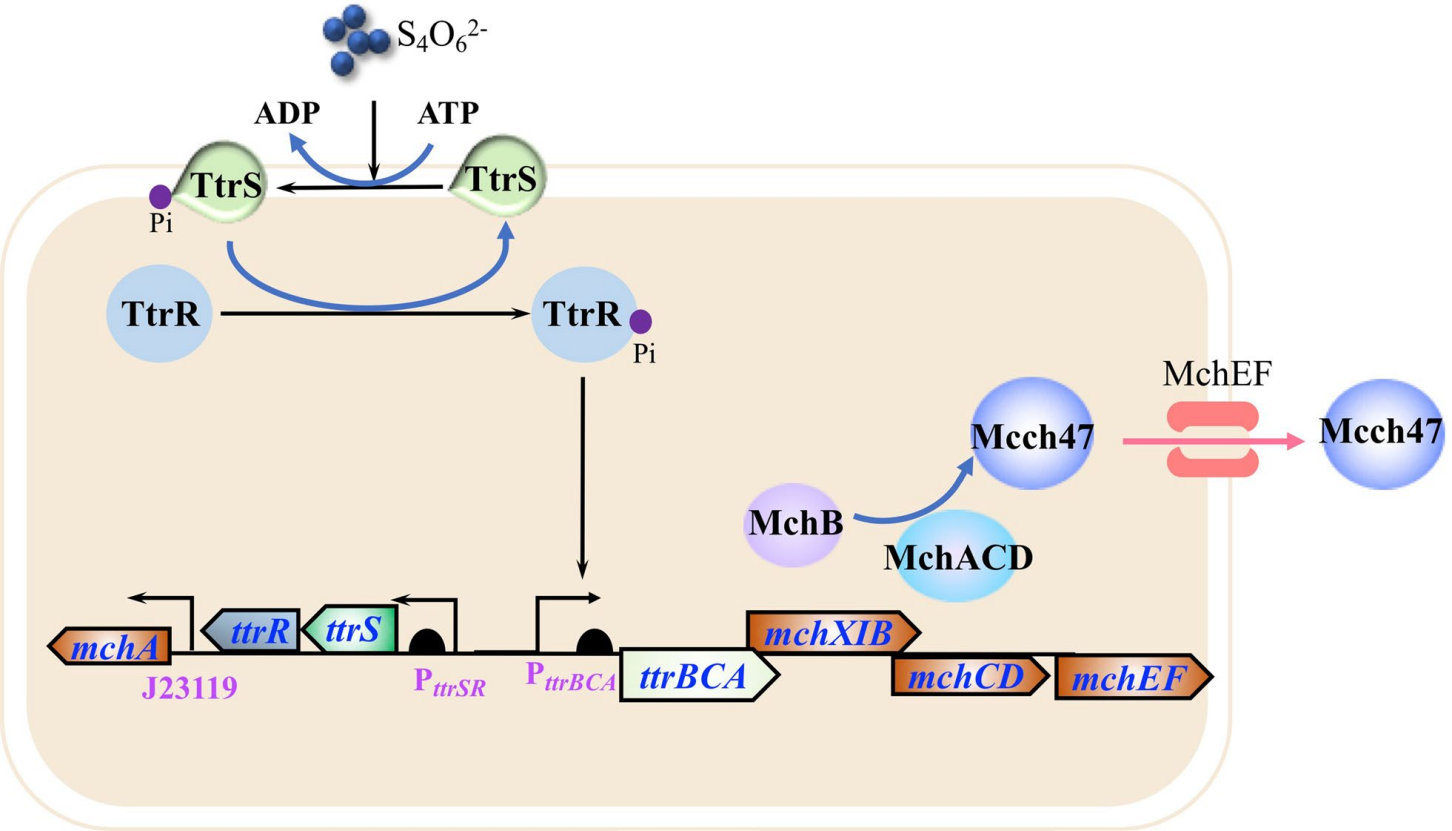
Engineering probiotic strain for the inhibition of *Salmonella* infection. The tetrathionate sensing system and Microcin H47 (Mcch47) production system were introduced into *E. coli* Nissle 1917. The *ttr* operon (*ttrRSBCA*) encodes tetrathionate reductase and the *mch* operon (*mchAXIBCDEF*) encodes Mcch47. During the course of *Salmonella* infection, tetrathionate was generated in the inflamed gut. In the presence of tetrathionate, the promoter P_ttrBCA_ was activated by the tetrathionate sensor histidine kinase TtrS and the response regulator TtrR. The transcription of the *mch* operon was initiated to produce Microcin H47 for the inhibition of *Salmonella*

*Salmonella* infections cause a variety of symptoms such as diarrhea, nausea, abdominal cramps and fever [65, 66]. *Salmonella* bacteria are often found in animals such as livestock, rodents and poultry, but sometimes it can spread to human in food contaminated by infected animal feces [65, 66]. Previously, a chromosome-encoded antibiotic microcin H47 was identified in *E. coli* that exhibited the inhibition effect on the growth of *Salmonella* [67]. On this basis, Sassone-Corsi et al. demonstrated that engineering of *E. coli* Nissle 1917 to produce microcin can therapeutically displace pathogen *S. enterica* in the inflamed gut under specific environmental conditions such as iron limitation [68]. It has been reported that reactive oxygen species produced during gut inflammation reacted with luminal thiosulfate, resulting in generation of tetrathionate [69]. In *Salmonella* species, the *ttr* operon (*ttrRSBCA*) is a locus encodes tetrathionate reductase for anaerobic respiration, which enable this pathogen with the ability to utilize the produced tetrathionate as an electron acceptor for respiration. As a result, the growth of pathogen *Salmonella* species outcompetes with intestinal microbiota during inflammation conditions [69]. Based on this mechanism, Palmer et al. developed a *E. coli* Nissle 1917 strain harboring both microcin H47 production system and tetrathionate sensing system to inhibit *Salmonella* infections (Fig. 2). In this strain, the production of microcin H47 was induced by the presence of tetrathionate, increasing the ability of the probiotic strain to inhibit overgrowth of infectious *Salmonella* species under inflammatory conditions [61]. MccJ25 is an antibacterial peptide microcin secreted by *E. coli* AY25. Mature MccJ25 can form a unique and stable lasso structure to inhibit transcription by bacteria RNA polymerase [70]. The probiotic *E. coli* Nissle 1917 was modified to express and secrete MccJ25 for the reduction of *S. enterica* in turkey gastrointestinal tract. The results shown that administration of the probiotic decreased the *Salmonella* carriage by 97% in the treated group and has no significant impact on the native microbiome in turkey ceca. It also observed that the modified probiotic demonstrated higher efficacy compared to treatment with traditional antibiotic enrofloxacin [71].

Cholera is an acute secretory diarrheal disease caused by infection of the intestine with the *Vibrio cholerae* [72, 73]. The pathogen *V. cholerae* can colonize in the small intestine and produces toxins that cause severe watery diarrhea. If untreated, severe dehydration can rapidly result in shock and even death in a short time [72, 73]. *V. cholerae* utilizes a sophisticated quorum sensing system to control its infection behavior in a density-dependent manner. At low cell density, the expression of virulence factors is initiated for establishment of infection. When the bacterial numbers are high, the expression of virulence factors is repressed by the high-level accumulation of autoinducers [74]. Based on this mechanism, Duan et al. engineered a probiotic *E. coli* strain to crosstalk with quorum sensing and thereby prevent *V. cholerae* from producing toxin. Administration of therapeutic probiotic strains led to an increased survival rate and decreased toxin formation in infant mouse models [75, 76]. In addition, Mao et al. designed and constructed a probiotic strain to sense *V. cholerae* in mouse gut. Oral administration of *L. lactis* could kill *V. cholerae* by secreting lactic acid in infant mouse cholera challenge models [77]. In another example, a live cholera vaccine candidate was created by genetically engineering a variant cholerae EI Tor strain, the modifications include remove of the essential virulence factors, reducing potential adverse reactions and prevention of the organism from becoming toxic again. It was demonstrated that this vaccine can effectively confer probiotic-like cholera protection in the short time in an infant rabbit model of cholera [78].

### Engineering probiotics for diagnosis and detection of diseases

**Fig. 3:**
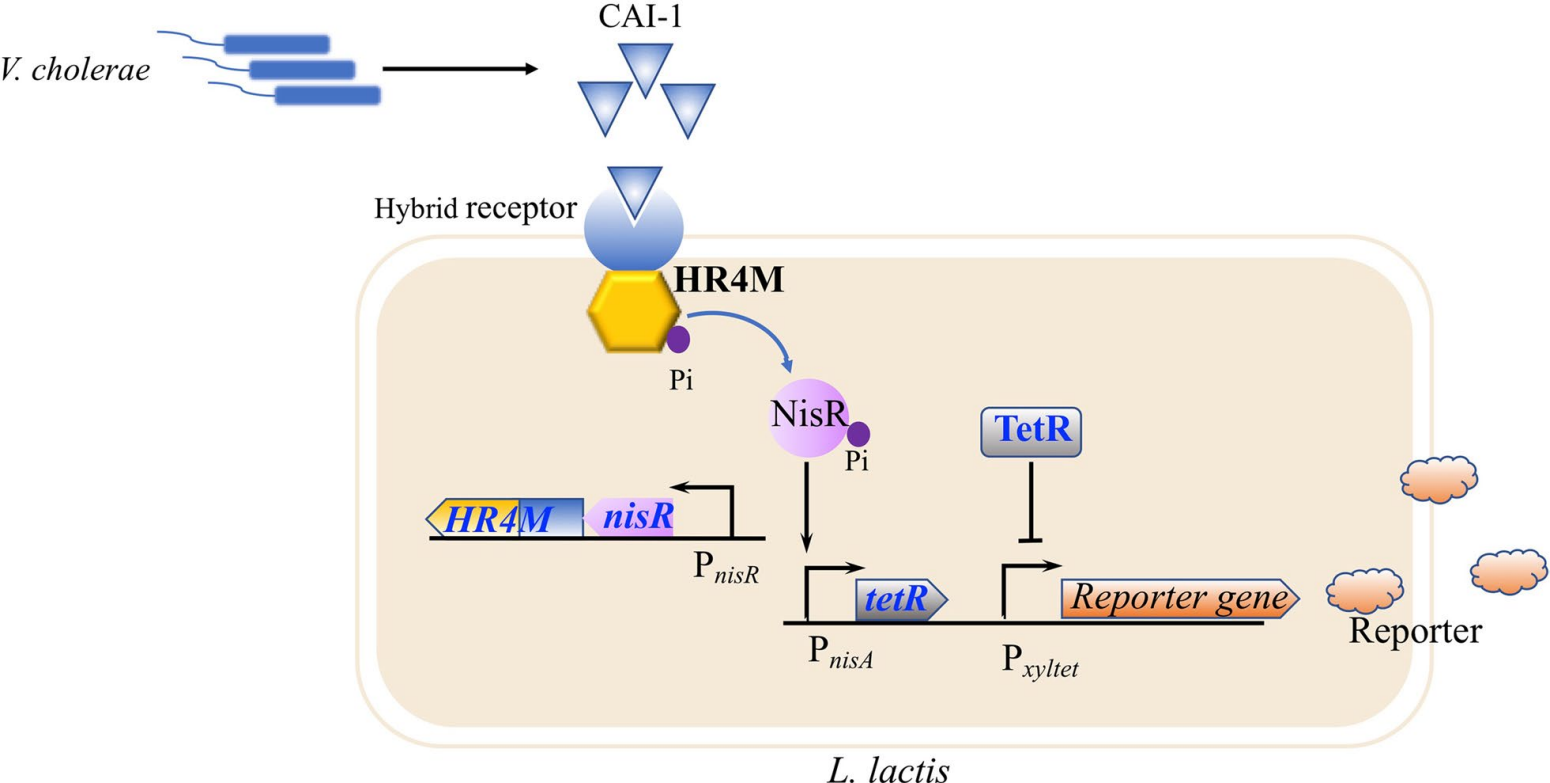
Engineering *L. lactis* probiotic strain for the detection of cholera infection. HR4M, a functional hybrid receptor variant was constructed by fusing transmembrane ligand binding domain of CqsS from *V. cholerae* with the signal transduction domain of NisK from *L. lactis*. The *V. cholerae*-sensing element consists of a HR4M-NisR two-component sensing system and a TetR-P_xyltet_ signaling system. In the absence of CAI-1, the phosphorylated NisR activates the expression of *tetR,* which is a transcriptional repressor for the promoter P_xyltet_, thus lead to inhibition of the reporter gene. In the presence of CAI-1, the HR4M-NisR two-component sensing system stops its phosphorelay, thus repressing the expression of *tetR* and resulting in the activation of the reporter gene

Another application of engineered probiotics is diagnosis and detection of diseases. Engineering of the bacteria to sense an important molecule in the body and then to produce a specific signal enables probiotics to become a diagnostic device. Introduction of the quorum sensing system into the probiotics is a commonly used strategy to sense the infected pathogens [77]. For instance, a diagnostic circuit in *L. lactis* was developed for in situ detection of a molecule produced by *V. cholerae*, thereby to get an early alert to cholera infection by the color changing of the host’s feces samples (Fig. 3). In this work, a novel signal molecule-sensor device was constructed by fusing the transmembrane ligand binding domain of CqsS from *V. cholerae* and the signal transduction domain of NisK from *L. lactis.* Once the engineered *L. lactis* detected the signaling molecule produced by *V. cholerae*, it will secrete the *β*-lactamase to demonstrate colorimetric shift in the presence of nitrocefin. Oral administration of the engineered *L. lactis* to cholera infected mice led to positive signals in their feces samples [77]. Formylated peptides are ubiquitous signal peptides produced by a broad range of bacterial species [79]. The human formyl peptide receptor FPR1 is able to monitor the presence of formyl peptides at nanomolar scale [80]. On this basis, Sedlmayer et al. constructed an artificial microbial-control circuit by coupling of formyl peptide sensor with the quorum sensing system to detect and destroy pathogens infection. Cells equipped with this system can detect formyl peptides generated by pathogens with high sensitivity and activate the production of the quorum sensing signaling molecule autoinducer-2 (AI-2) to efficiently reduce the biofilm formation [81].

Except for recognition of pathogen infections, the sensor-equipped probiotics could also be used to produce detectable signals for cancer diagnosis. The oxygen-limited and necrotic regions of tumors are attractive environments for some anaerobic bacteria such as *E. coli*, *Clostridium* and *Salmonella* [82–84]. The natural propensity to colonize tumor environments enables those bacteria to selectively target tumors and metastases [84]. Danino et al. engineered probiotic *E. coli* Nissle 1917 as a diagnostic tool to detect liver metastasis in mice by specifically colonizing liver tumor and producing a detectable signal in urine. In this design, the expression cassettes *luxCDABE* (encoding uciferase) and *lacZ* (encoding *β*-galactosidase) were co-expressed in *E. coli*, which enabled the created probiotic strain PROP-Z to generate luminescent signal and colorimetric readout [85, 86]. The murine model of liver metastasis was treated by oral administration of PROP-Z and intraperitoneal injection of D-luciferin, it was observed a good specificity and correlation between tumor diameters and signal radiances. To further enhance the stability and efficacy of this system, a toxin-antitoxin system and the gene *dlp7* from *Bacillus subtilis* were introduced into PROP-Z to force the cell to maintain the plasmid and equally segregate it upon cell division. Oral delivery combined with the administration of luciferin/galactose conjugate resulted in the release of luciferin into circulation, which can be cleared by the kidney allowing for detection in murine urine samples [87]. In another example, Kotula et al. constructed a toggle switch in *E. coli* that enables detection, memory and recording of exposure to antibiotics within the gut environment. Especially, this system consists of a stable memory element based on the phage lamba *cI/cro* genetic switch and a trigger device in which the expression of the gene *cro* is controlled by a tetracycline-inducible promoter. The presence of the tetracycline switches the memory element from the cI state into the Cro state and triggers the expression of a *β*-galactosidase reporter gene (encoded by *lacZ)* within the memory element. Oral administration of the engineered *E. coli* strain to tetracycline-exposed mice enables recording of the event in the mouse gut by analyzing the fecal samples [88].

**Fig. 4:**
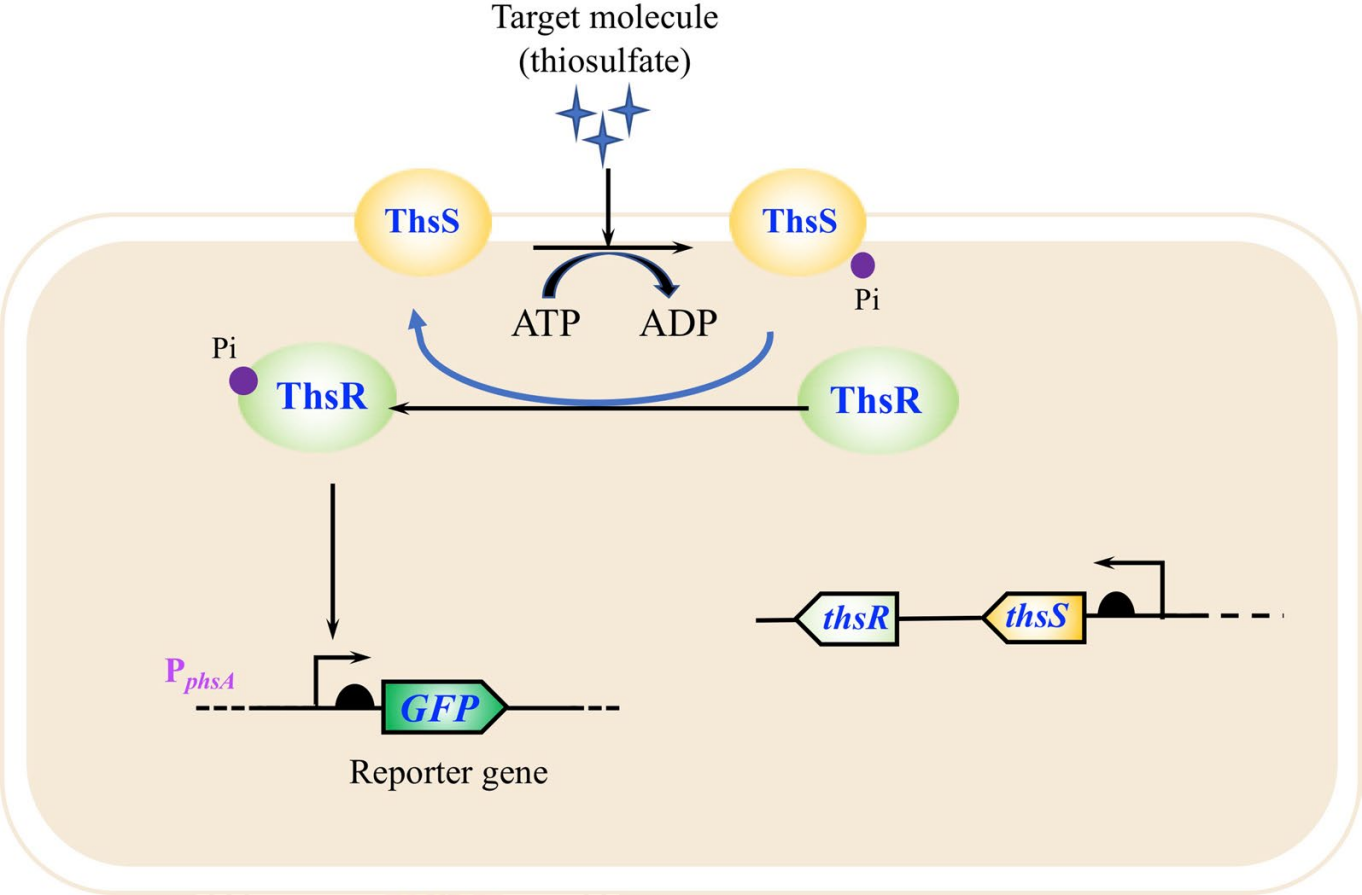
Engineering probiotic *E. coli* strain for the detection of gut inflammation via a thiosulfate sensor. The presence of thiosulfate initiates the phosphorylation of thiosulfate response regulator ThsR and sensor ThsS, which activates the transcription of promoter P_*phsA*_. Over-expression of a fluorescent reporter gene under control of the promoter P_*phsA*_, the engineered probiotic *E. coli* strain harboring thiosulfate sensor demonstrated specific response and fluorescence in colon inflammation mouse model

Probiotics could also be engineered to detect inflammation via small-molecule sensing. Nitric oxide (NO) is a signaling molecule that can be generated by many cell types [89]. It is recognized as an anti-inflammatory compound under normal physiological conditions [90]. While, over-production of NO under abnormal situations causes tissue damage, inflammation and even cancer [91]. Thus, the high level of NO serves as a proinflammatory indicator in inflammatory bowel diseases. Archer et al. constructed a living bacterial sensing device to detect NO for marker of gut inflammation. The engineered *E. coli* strain equipped with this synthetic device perceived NO production in the gut. The presence of the NO activated the expression of a DNA recombinase, causing permanent activation of a DNA switch that is inherited by the progeny after cell division [92]. Another interesting small molecule is tetrathionate. Sulfate reducing bacteria (SRB) in the colon can produce hydrogen sulfide, which is extremely cytotoxic to the host cells [93, 94]. High level of hydrogen sulfide inactivates cytochrome c oxidase and inhibits the oxidation of butyrate in colon epithelial cells [95]. In the host, hydrogen sulfide can be detoxicated to thiosulfate, which is further oxidized to tetrathionate by reactive oxygen species (ROS) during gut inflammation [69, 96]. Hence, tetrathionate could be used as a biomarker for detection of gastrointestinal tract disease if this molecule can be precisely sensed in the host. It has been reported that some pathogenic bacteria are capable of using tetrathionate as a terminal electron acceptor to obtain a growth advantage. Those bacteria possess a two-component regulatory system (TCRS), which is able to specifically sense the presence of tetrathionate and sequentially reduce its accumulation by activating the expression level of tetrathionate consumption pathways [69, 96]. Based on this mechanism, Riglar et al. developed a probiotic *E. coli* strain that can sense and remember tetrathionate exposure in the gut by employing the TCRS from *Salmonella* and engineered this system to activate a phage lamba *cI/Cro* genetic element [97]. In another study, Daeffler et al. identified novel TCRS homologs from marine *Shewanella* species, which includes a thiosulfate sensor regulator ThsSR and a tetrathionate sensor regulator TtrSR (Fig. 4). It was found that both sensors functioned well in the complex colonic environment. With a fluorescent reporter gene inserted, the engineered probiotic *E. coli* strain harboring thiosulfate sensor demonstrated specific response and fluorescence in colon inflammation mouse model [98].

## Conclusions

The recent studies present an increasing evidence indicating the nice healthy effect of probiotics on an individual [99–101]. Utilization of probiotics as dietary management for treatment of diseases has gained much attention in food and medicine industries [102]. The development of metabolic engineering and synthetic biology enable disclosing their mechanism of action and creating novel probiotic strains with desired functions. Engineering of probiotic strains for detection and treatment of metabolic disorders, inflammations and pathogen infections has made great progress in recent years [28, 54]. Those advancements enable us to provide insights into the principles and limitations for development of perfect probiotics for human health.

Metabolic disorders including hyperammonemia and phenylketonuria are usually caused by excessive accumulation of the toxic products or insufficient supply of the essential metabolic intermediates [26, 44]. Engineering probiotics harboring supplemental enzymes or pathways to supply essential intermediates or remove toxic compounds serves as a promising approach for the treatment of metabolic disorders caused by the enzyme deficiency. However, the specificity and activity of the introduced enzymes should be carefully evaluated to avoid resulting in other unnecessary metabolic disorders. Currently, there are a lot of strains used as probiotic hosts including *E. coli* Nissle 1917, *L. lactis*, *B. subtilis, Saccharomyces cerevisiae, Enterococcus* and *Streptococcus* species [77, 92, 103, 104]. In some cases, those microbes are genetically modified for a specific effect, which presents a major limiting-factor for their large-scale applications. It is well known that some people believe those genetically modified microorganisms are dangerous to their health. Additionally, it is necessary and essential to conduct safety assessments on those probiotic strains for human use, including the short-term, long-term side effects and potential vulnerability or pathogenicity to the consumer or patient. Engineering probiotics also can be used to inhibit pathogen infections by secreting antimicrobial peptides or bacteriocins that enable probiotic strains to obtain growth advantage [62, 69]. However, like antibiotics, most of the antimicrobial peptides or bacteriocins are not specific. Use of those chemicals may cause intestinal dysbiosis, metabolic disorders and other side effects. In addition, those antimicrobial peptides or bacteriocins are usually toxic to the probiotic strains and even kill the producing cells. In some cases, those orally administrated antimicrobial peptides or bacteriocins are rapidly recognized by the immune system and degraded before they can reach the target site of infection. Engineering probiotics for detection and diagnosis of diseases such as inflammatory bowel disease and pre-symptomatic abnormal changes are involved in sensing an important molecule which can be quorum sensing signaling molecules or inflammatory environmental conditions, and then producing some signals that can be detected in the urine or feces [77, 87]. Such in vivo monitoring method is able to track the gut health status in patients without more invasive tests, such as endoscopy. However, several issues still need to be addressed for widely application of those diagnostic devices. The interface between the probiotic strains and host cells is difficult to understand. Besides, the sensitivity and specificity of the engineered sensors are hardly to control. Thus, most of the current studies have not yet advanced into clinical trials. Despite those limitations, it is still need to pay a lot of attention on engineering probiotic strains for human health improvement. We believe that the increasing knowledge of the human microbiome and the mechanisms of disease, the fast development of metabolic engineering, synthetic biology and other disciplines will facilitate optimization of strategies in design and construction of robust and effective probiotics to prevent disease and improve human health.

## Data Availability

Not applicable.

## Abbreviations

IBD: Inflammatory bowel disease
T1D: Type I diabetes
T2D: Type II diabetes
IL-10: Interleukin-10
NOD: Non-obese diabetic
GLP: Glucagon-like peptide
HIS: Polyhistidine
ZDF: Zucker diabetic fatty
PKU: Phenylketonuria
PAH: Phenylalanine hydroxylase
PAL: Phenylalanine ammonia lyase
LAAD: l-amino acid deaminase
ASF: Altered schaedler flora
BBB: Blood–brain-barrier
AADC: Aromatic acid decarboxylase
TyrDC: Tyrosine decarboxylase
AFMT: *S*-α-fluoromethyltyrosine
AHL: *N*-acyl-homoserine lactone
EHEC: Enterohemorrhagic *E. coli* strain
AI-2: Autoinducer-2
NO: Nitric oxide
SRB: Sulfate reducing bacteria
TCRS: Two-component regulatory system
